# Bacterial Synergism in Breast Necrotizing Fasciitis: A Case Report on Diagnostic Dilemmas, Therapeutic Challenges, and Reconstructive Management

**DOI:** 10.1155/crdi/3731779

**Published:** 2025-08-07

**Authors:** Leila Essid, Leslie Ann See, Georges Tarris, Narcisse Zwetyenga, Vivien Moris

**Affiliations:** ^1^Department of Plastic Reconstructive and Hand Surgery, Department of Oral and Maxillofacial Surgery, Dijon University Hospital, Boulevard de Lattre de Tassigny, Dijon F-21000, France; ^2^Department of Pathology, University Hospital François Mitterrand of Dijon-Bourgogne, Dijon, France; ^3^Department of Maxillofacial, Plastic-Reconstructive, and Aesthetic Surgery, Hand Surgery Unit, Dijon University Hospital, Boulevard Maréchal-de-Lattre-de-Tassigny, Dijon F-21000, France; ^4^Lipids, Nutrition, and Cancer Team, NuTox UMR866, University of Burgundy Franche-Comté, Boulevard Jeanne-d'Arc, Dijon F-21000, France

## Abstract

**Introduction:** Necrotizing fasciitis (NF) is a rapidly progressive, life-threatening soft tissue infection that primarily involves the fascia and subcutaneous tissues. While it typically affects the extremities, perineum, or trunk, NF of the breast remains an exceptionally rare and underdiagnosed entity, often leading to delayed intervention and high morbidity.

**Case Presentation:** We report the case of a 57-year-old woman with poorly controlled Type 2 diabetes who presented to the emergency department with diabetic ketoacidosis and erythema of the left breast. Initial arterial blood gas analysis revealed profound metabolic acidosis (pH 6.89, PaCO_2_ 12.8 mmHg, bicarbonate 2.5 mmol/L, and base excess −31.5 mmol/L). Despite initial antibiotic therapy, the breast symptoms worsened, raising concern for inflammatory breast cancer. Imaging revealed subcutaneous emphysema and extensive soft tissue inflammation. A diagnosis of NF was confirmed, prompting emergency surgical intervention. A left mastectomy was performed, with resection of necrotic fascia and pectoralis major. Microbiological cultures identified a polymicrobial infection with *Escherichia coli*, *Citrobacter*, and *Actinotignum schaalii*. The patient received targeted antibiotic therapy and supportive care, including pain management and fluid–electrolyte balance. Reconstruction was initiated 8 months later with contralateral breast reduction and fat grafting.

**Discussion:** Breast NF poses significant diagnostic challenges due to the organ's unique vascular anatomy and the rarity of the condition. Delays in diagnosis can be fatal. This case underscores the importance of clinical vigilance, prompt imaging, and early surgical debridement. The synergistic effect of polymicrobial infections was evident in the rapid progression. Reconstruction remains an essential component of care, with satisfactory outcomes achievable through fat grafting and symmetry procedures.

**Conclusion:** Breast NF requires rapid diagnosis and aggressive multidisciplinary management. This case illustrates the need for increased awareness to reduce diagnostic delays and improve survival and reconstructive outcomes.

## 1. Introduction

Necrotizing fasciitis (NF) is a severe, life-threatening bacterial infection that primarily affects the fascia, the connective tissue that surrounds muscles, nerves, fat, and blood vessels. It is defined by its rapid progression and aggressive tissue destruction, leading to widespread necrosis of subcutaneous tissue and fascia, often with relative sparing of overlying skin and underlying muscle in the early stages [1].

A defining feature of NF is its fulminant course, where bacteria, often in polymicrobial synergy or as monomicrobial pathogens such as *Streptococcus pyogenes*, produce toxins and enzymes that disrupt tissue planes and blood supply, resulting in rapid tissue breakdown and systemic toxicity [2]. NF is often misdiagnosed in its early phase due to nonspecific symptoms, but it quickly evolves to include severe pain out of proportion to clinical findings, swelling, erythema, and signs of systemic inflammation. Without urgent surgical debridement and broad-spectrum antibiotics, NF can lead to septic shock, multiorgan failure, and death, underscoring its classification as a true medical and surgical emergency [3]. Although NF most commonly involves the extremities, abdominal wall, and perineum, its occurrence in the breast is exceptionally rare and underrecognized in clinical practice [1]. The rarity of breast NF, combined with its atypical presentation, frequently leads to diagnostic delays that can significantly impact patient outcomes.

Preexisting conditions such as diabetes mellitus, immunosuppression, and vascular disease are well-established risk factors for NF [2]. However, the breast's unique anatomical structure with a robust blood supply and thick tissue layers can mask early signs of infection, further complicating the diagnostic process. In the majority of reported cases, breast NF has been associated with recent surgical procedures or traumatic events [3], while spontaneous like in this case remains exceedingly uncommon.

We are presenting the case of a 57-year-old diabetic woman whose clinical history started with an abscess of the left breast, initially treated with antibiotics, related to an unbalanced diabetes.

## 2. Case Presentation

A 57-year-old woman with a history of Type 2 diabetes was admitted to the emergency department, presenting with severe diabetic ketoacidosis and erythema of the left breast. Arterial blood gas analysis revealed profound metabolic acidosis, with a pH of 6.89, PaCO_2_ of 12.8 mmHg, bicarbonate level of 2.5 mmol/L, and a base excess of −31.5 mmol/L. Following intensive medical management, acid–base parameters normalized, with a pH of 7.40, bicarbonate at 23.9 mmol/L, and a base excess of −0.3 mmol/L (Table 1).

Additional laboratory investigations showed a blood glucose level of 39.51 mmol/L and a ketonemia of 7.7 mmol/L. Urinalysis revealed the presence of blood, ketones, glucose, and proteins, with a pH of 6. Blood lactate was measured at 1.6 mmol/L.

Her medical history included untreated rheumatoid arthritis, hepatic steatosis, smoking cessation in 2016 (40 pack-years), and multiple allergies.

She was initially admitted for stabilization of her diabetes with NovoRapid 100 U/mL at 8 units/hour via continuous infusion, along with paracetamol 1 g and a hydration protocol as follows: 0.9% NaCl, 1 L over 1 h, followed by 1 L over 2 h, then another 1 L over 2 h, and finally 250 mL of 1.4% sodium bicarbonate over 1 h.

Concerning the left breast erythema, an intravenous (IV) antibiotic treatment with clindamycin 600 mg every 8 h was initiated. Over the following 5 days, swelling, redness, and pain in the left breast worsened, accompanied by tachycardia and fever. Laboratory tests revealed inflammatory markers, including a white blood cell count of 16,000 and a C-reactive protein (CRP) level of 217.

Clinically, a swollen area was observed in the periareolar region and the inframammary fold, with a necrotic area involving the inferior internal and external quadrants. No subdermal crepitus was detected.

Given the clinical findings, a differential diagnosis of inflammatory breast cancer was considered. A biopsy under ultrasound guidance was performed. It is worth noting that the patient had undergone routine checkups, including a mammogram, just 1 month prior.

**Figure 1:**
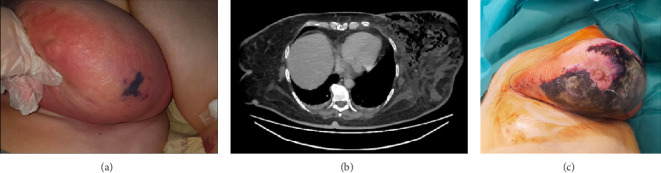
(a) Initial presentation at the ER showing swelling, redness, and a small necrotic area in the inferior internal quadrant of the breast. (b) Thoracic CT scan revealing extensive subcutaneous emphysema. (c) Progression of necrosis affecting the entire breast.

As symptoms progressed, a chest CT scan revealed widespread soft tissue inflammation of the left breast and extensive subcutaneous emphysema (Figure 1).

Based on these clinical and imaging findings, NF of the breast was diagnosed. A left mastectomy was performed, which revealed partial necrosis of the fascia and pectoralis major. The area of necrosis extended to the nipple and lower breast quadrants. Necrotic tissue was excised until viable, bleeding tissue was observed, and microbiological swabs were taken. The surgical site was irrigated extensively with saline solution and hydrogen peroxide.

**Figure 2:**
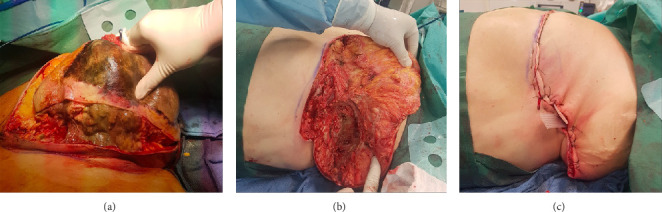
(a) Preoperative aspect of the breast FN. (b) Extension of the necrosis. (c) Immediate postoperative aspect.

At the end of the procedure, a large external defect remained, and two internal Delbet drains were placed (Figure 2).

**Figure 3:**
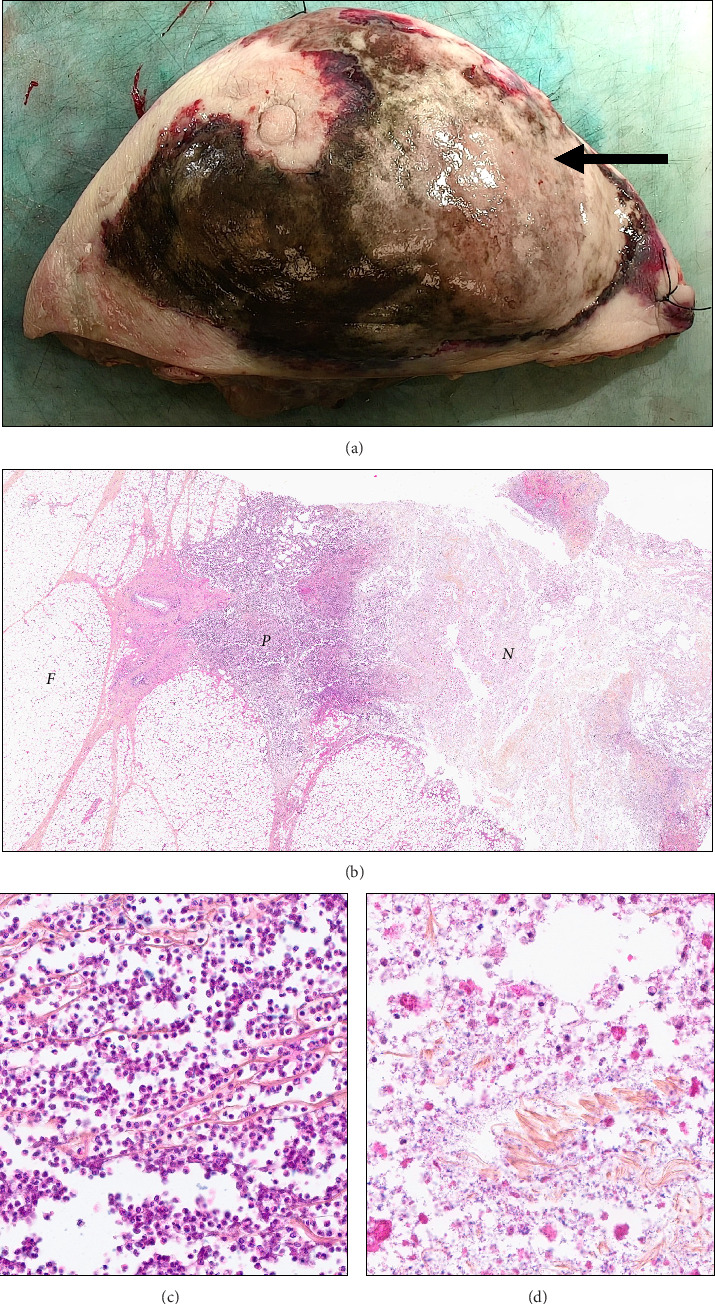
Pathological assessment of the breast surgical resection specimen. (a) Gross examination of the surgical sample revealed periareolar subcutaneous necrosis and pus (arrow). (b) Histopathological examination of the sample revealed necrotizing fasciitis, characterized by liquefactive necrosis and pus (P), with numerous neutrophils infiltrating the fat tissue (F), and areas of coagulative necrosis (N) at low magnification (HES, X100), thus confirmed at higher magnification for (c) neutrophilic infiltration (X400) and (d) coagulative necrosis (X400).

Broad-spectrum antibiotic therapy with linezolid was initiated and subsequently adjusted based on microbiological findings, which identified *Escherichia coli*, *Citrobacter*, and *Actinotignum schaalii*. The patient was then treated with metronidazole 500 mg IV every 8 h and levofloxacin 500 mg IV once daily for 10 days. The clinical evolution was favorable, with a significant reduction of the external defect within 10 days. The anatomopathological examination (Figure 3) confirmed suppurative inflammation and ulceration consistent with NF.

Concerning the pain management, the patient was started on sustained-release morphine sulfate (Skenan LP) at a dose of 10 mg twice daily for baseline analgesia. For breakthrough pain, immediate-release morphine sulfate (Actiskenan) was prescribed at a dose of 3 mg, to be taken as needed, up to every 4 h.

IV hydration was administered using 5% glucose at 2 L per 24 h, with each liter supplemented by 4 g of potassium chloride, under close electrolyte monitoring.

The patient achieved good scar healing and complete skin closure 8 months after surgery. She was then considered for breast reconstruction. Ten months postsurgery, she underwent right breast reduction and liposculpture fat grafting to the left breast.

**Figure 4:**
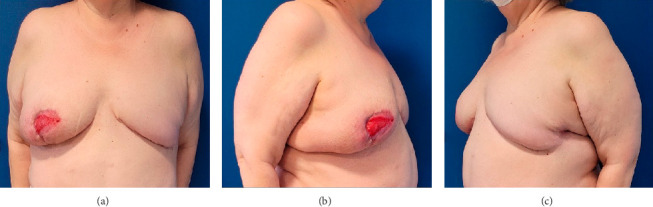
Result at 3 months after the reconstructive surgery. (a) Frontal view. (b) Right oblique view. (c) Left oblique view.

Postreconstruction recovery was marked by partial necrosis of the nipple and suboptimal fat graft retention. Despite these complications, the overall outcome was satisfactory (Figure 4).

## 3. Discussion

NF is a life-threatening infection that involves the fascia and subcutaneous tissues. It spreads rapidly and most commonly affects the extremities, trunk, and perineum [4]. In France, the annual incidence of NF is relatively low, estimated at 2–4 cases per 100,000 inhabitants [5].

Breast fasciitis remains a particularly rare entity. A 20-year literature review reported only 40 documented cases [6], with frequent misdiagnoses contributing to a general mortality rate of 18.7% (3 out of 16 cases) [7].

Diagnosing NF is challenging and primarily relies on clinical examination, which typically reveals erythema, swelling, pain, and the presence of necrotic areas with poor vascularization [8, 9]. Diagnosing NF of the breast is even more difficult due to the thickness of breast tissues and their robust blood supply, which can delay visible cutaneous reactions. This often leads to misdiagnosis as cellulitis, mastitis, a breast abscess, or even inflammatory breast cancer [8, 10–12].

Several triggering factors have been identified in the literature, including minor trauma and prior surgical procedures such as mammoplasty, tumor excision, and stereotactic biopsy. However, NF can also occur spontaneously, accounting for 52.5% of cases, as noted in the review by Cai et al. [6].

Predisposing comorbidities may exist as diabetes, obesity, immunodepression, like in our case, where the patient suffered from an unbalanced diabetes, obesity, and an unfollowed rheumatism.

A large choice of imaging has been used to diagnose the breast NF from CT scans to MRI [13], but none of them must delay the surgical debridement. In this case, the patient had undergone a CT scan that showed abnormal fluid and gas along the fascia plane of the breast.

As soon as the NF diagnosis is suspected, an urgent surgical debridement must be set.

Any delay in the surgical management can lead to the patient's death [6].

The surgical management of NF is well-defined: In most reported cases, patients undergo extensive debridement or radical mastectomy to achieve complete resolution. A second-look surgery is performed if there is evidence of secondary necrosis extension [14].

In some cases, hyperbaric oxygen therapy has been employed for its antimicrobial effects, and vacuum-assisted closure has been used to promote the growth of healthy soft tissues. [15–17].

Two types of NF are described in the literature based on the microbiological profile. Type I results from polymicrobial infections involving both anaerobic and aerobic organisms, while Type II is caused by a monomicrobial infection, typically *Streptococcus pyogenes*. In our case, the infection was polymicrobial, involving *Escherichia coli*, *Actinotignum schaalii*, and *Citrobacter* species.

Polymicrobial infections, such as those involving *Escherichia coli*, *Citrobacter*, and *Actinotignum schaalii*, play a crucial role in the rapid progression of NF due to their synergistic effects. Aerobic bacteria such as *E. coli* and *Citrobacter* produce destructive toxins and enzymes (e.g., hemolysins and collagenases) that promote local inflammation and tissue destruction while creating a low-oxygen environment. These conditions allow anaerobic bacteria such as *A. schaalii* to thrive, further exacerbating tissue necrosis by producing gases and worsening local hypoxia [18]. This synergy creates a feedback loop where bacteria exploit weakened immune defenses to spread deeper into tissues, causing extensive and challenging-to-manage damage [10].

Reconstruction was considered once the acute phase had resolved. The literature describes several options, including secondary healing, skin grafting, latissimus dorsi flaps, free flaps such as DIEP, breast silicone implants, and fat transfer [14]. For our patient, secondary reconstruction was performed using fat transfer to the affected breast, combined with contralateral breast reduction.

## 4. Conclusion

NF of the breast is a rare but life-threatening condition that demands prompt diagnosis and aggressive management. This case highlights the complexities in identifying and treating breast NF, emphasizing the importance of a multidisciplinary approach. Early surgical debridement, tailored antibiotic therapy, and supportive care are critical for improving patient outcomes. Polymicrobial infections, as observed in our patient, illustrate the synergistic interactions between aerobic and anaerobic organisms, further complicating the disease progression.

Reconstruction, once the infection has resolved, plays a crucial role in restoring functionality and aesthetics. Various reconstructive options, such as fat transfer and contralateral breast reduction, can provide satisfactory results, as demonstrated in this case. This report underscores the need for heightened clinical awareness of breast NF to ensure timely intervention and optimize long-term outcomes.

## Figures and Tables

**Table 1:** Biological and gasometric parameters: admission, surgery day, and postoperative follow-up.

	Emergency department admission	Surgery day	Complete healing (1-month postop)
Hb	14.7	8.2	13.9
Leukocytes	30.4	20.9	7.3
PNN	23.7	17.15	4.02
Lymphocytes	12	2.27	2.38
Monocytes	8	1.25	0.54
CRP	134	128	14.4
Blood sugar	39.51 mmol/L	10.4 mmol/L	7.05 mmol/L
pH	6891	7398	
PaCO_2_ (mmHg)	12.8	39.6	
Bicarbonate (mmol/L)	2.5	23.9	
Bicar standards (mmol/L)	5.2	24.2	
Base excess (mmol/L)	−31.5	−0.3	
Total CO_2_ (mmol/L)	2.9	25.1	

## Data Availability

The data that support the findings of this study are available on request from the corresponding author.
